# Infections

**Published:** 1916-11

**Authors:** 


### ABSTRACTS

Have you ever felt that this department of the BUFFALO MEDICAL JOURNAL was lacking in abstracts of your specialty, or that such as were published lacked the expert judgement which you yourself could have exercised in selection and preparation? If not, you are more charitable than the editor has been to himself. If so, will you assist in abstracting from a few journals, and thus make this department more representative and more valuable? Incidentally, there are few forms of study more helpful to the worker than this.

---

### Infections.

**Localized Tetanus.** E. Meriel, Soc. de Chir., Feb. 2, reports a case consisting of spasmodic monoplegia in a leg and one limited to the head without facial paralysis. The attenuation of virulence is ascribed to the prophylactic injection of antitoxin.

